# Incidence, Risk Factors and Causes of Severe Neonatal Hyperbilirubinemia in the South of Iran (Fars Province)

**DOI:** 10.5812/ircmj.3337

**Published:** 2013-03-05

**Authors:** Khadije Sadat Najib, Forough Saki, Fariba Hemmati, Soroor Inaloo

**Affiliations:** 1 Namazi Hospital, Shiraz University of medical sciences, Shiraz, IR Iran

**Keywords:** Hyperbilirubinemia, Neonatal, Causality, Risk Factors

## Abstract

**Background:**

Today, Severe hyperbilirubinemia is the most common cause of neonatal readmissions. Identification of the cause of neonatal hyperbilirubinemia is useful in determining whether therapeutic interventions can prevent severe hyperbilirubinemia.

**Objectives:**

We conducted this study to estimate the incidence of severe hyperbilirubinemia in Fars province and to determine the underlying causes and risk factors, which would be of value in identifying and implementing strategies to prevent morbidity from this condition.

**Patients and Methods:**

All infants less than 28 days referred due to severe indirect hyperbilirubinemia were included. Complete history, physical examination and lab work up were performed. This is a longitudinal prospective study in 2009-2010.

**Results:**

More common causes of severe indirect hyperbilirubinemia were blood group incompatibility, G6PD deficiency, sepsis and unknown. Risk factors of severe hyperbilirubinemia were Male sex, previous siblings with severe hyperbilirubinemia, early discharge, NVD, Breast feeding and cultural background of mothers.

**Conclusions:**

Our study showed severe neonate indirect hyperbilirubinemia is still prevalence in Fars province and ethnic and cultural background of the mothers was more effective than school education in preventing hyperbilirubinemia complication.

## 1. Background

Today, in North America severe hyperbilirubinemia is the most common cause of neonatal readmission (1-5). Identifying newborns at risk of clinically significant hyperbilirubinemia is important before they are discharged from Hospital (6-9). Icter or Jaundice is common event that seen in 60% of term neonate & 80% of preterm ones at birth, and it is often benign hyperbilirubinemia with a total bilirubin more than 95% percentile on the hour-specific. Bhutani monograms is accompanied with a high risk of bilirubin- induced neurologic dysfunction (BIND) (10). Acute bilirubin encephalopathy means the acute manifestations of BIND. Kernicterus means chronic and permanent sequel of BIND. Appropriate intervention such as phototherapy and exchange transfusion is used in every infant with severe hyperbilirubinemia. Even if these infants are adequately treated, long-term neurologic sequel (Kernicterus) cannot be prevented. Identification of the cause of neonatal hyperbilirubinemia is useful in determining whether therapeutic interventions can prevent severe hyperbilirubinemia. The most common causes of pathologic indirect hyperbilirubinemia are : 1) increased bilirubin production due to hemolytic disease process that includes the Immune mediated hemolysis (e.g. ABO or Rh incompatibility), inherited red cell membrane defects (e.g., hereditary spherocytoses and elliptocytosis), erythrocyte enzymatic defects (e.g., Glucose-6-phosphate dehydrogenase (G6PD) deficiency & pyrovate kinase deficiency) and sepsis. 2) Decreased clearance such as inherited defects in uridine diphosphogluconurate glucuronosyltransferase (UGT) (eg Crigler-Najjar syndrome and Gilbert’s syndrome) and 3) Increased entrohepatic circulation such as breast feeding failure jaundice and breast milk jaundice (10-15).

## 2. Objectives

We conducted this study to estimate the incidence of severe hyperbilirubinemia in Fars province and to determine the underlying causes and risk factors, which would be of value in identifying and implementing strategies to prevent morbidity from this condition.

## 3. Patients and Methods

This prospective cross sectional study had been performed on all infants less than 28 days referred to Namazi Hospital from February 2009 to February 2010 due to severe indirect hyperbilirubinemia (Bilirubin more than 95 percentile on the hour-specific Bhutani nomogram (10). They need intensive phototherapy or exchange, transfusion. History including birth weight, level of education of their mother, mother’s age, mother’s knowledge about the effect of hyperbilirubinemia on neonate, the onset time of hyperbilirubinemia, the onset of breast feeding, history of formula feeding, history of intravenous oxytocin infusion during labor, technique of delivery, time of meconium passage, times of first medical visit of neonate, the family history of jaundice & their cause in other siblings were taken from all mothers. Complete physical examination including weight on admission, cephal hematoma, level of consciousness, signs of Kernicterus (e.g. opistotonus, and convulsion) were performed carefully. Lab data including complete blood count (CBC), reticulocytes count (RC total and direct serum bilirubin, direct combs' test, G6PD level, urine and blood culture, blood group & Rh type of mother and neonate and c-reactive protein (CRP) were taken in all these neonate to define the cause of hyperbilirubinemia. Bilirubinemia tested with Diazo method, and G6PD level was checked with spot florescence method. Statistical analysis was done with SPSS 11.5 software with using chi-square test and T-test. Risk factors are concluded from comparison between the mean level of bilirubin between two groups of male or female, NVD or Cesarian section delivery, Breast feeding or formula feeding, had or had not history of previous sibling with severe huperbilirubinemia, had or had not concept of using herbal medicine instead of referring to Doctor when neonate had Icter by using pair T test. This study was in accordance with the ethical principles of the Helsinki II declaration. The study protocol was approved by the local ethics committee in Shiraz medical university, Department of medical ethics. All parents gave their informed written consent.

## 4. Results

**Figure 1.**
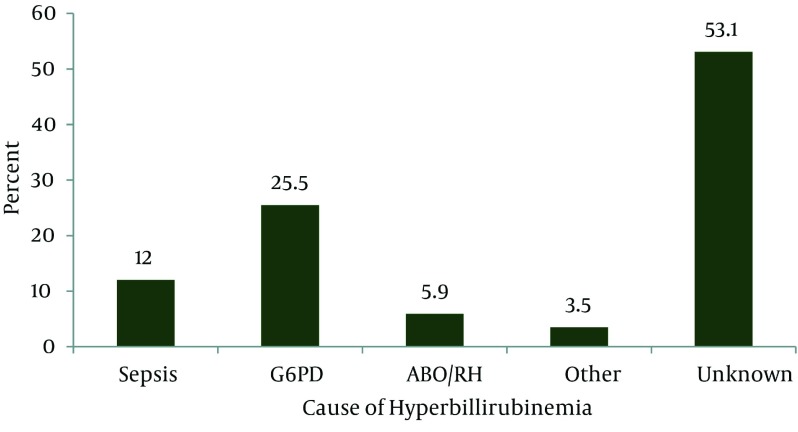
Cause of Hyperbillirubinemia in Neonates

From 1134 neonates with indirect hyperbilirubinemia referred to Namazi hospital from February 2009 to February 2010, 170 neonates were included in this study according to inclusion & exclusion criteria. Ninety nine of them (58.2%) were male and 71 (41.8%) were female, 125 neonate (73.5%) were delivered with normal vaginal method (NVD) and 45 neonate (26.5%) with cesarean section method, 66 neonate (32.4%) used oxytocin during labor. Birth weight of neonates was 3068 (526) g (Min: 1550 g, Max: 4300gr). 19 neonate (11.4) developed jaundice in first 24 hours after birth. 137 neonate (73.5%) developed jaundice after discharging from hospital, 153 mothers (90.3) had known that they should have referred to doctor if the neonate had been yellowish & 118 of mothers (69.4%) also knew that jaundice may cause brain damage. The age of 90 mothers (52.6%) was more than 25 years old, and 93 mothers (55%) were high graduates. Time of first feeding of neonate was 3.99 (9.99) hours after birth (Min: 0.5 hour, Max: 72 hours). One hundred fifty four neonates had exclusive breast feeding, 3 neonates had exclusive formula feeding and 12 neonates had both breast and formula feeding. Neonates passed meconium 10.9(10.06) hours after birth (Min: 0, Max; 48). 46 neonates (27.9%) had history of jaundice in their siblings (12.4% of need phototherapy and 5.3% of them need exchange phototherapy). Cause of jaundice in the siblings were sepsis (11.4%), G6PD deficiency (30.7%), ABO and Rh incompatibility was 5.8% and unknown (50%) (Table 1). Bilirubin level on admission was 2.059(5.81) mg/dl (Max: 42, Min: 9.5). 8 neonate (4.7%) had cephal hematoma and 9 neonate (5.4%) had cyanosis on admission. Thirty three neonates (18.8%) had poor feeding irritability and letharginess on admission when started 4.65(6.58) hours after birth (Min, 1hr, Max, 24 hours). Two neonates had signs of Kernicterus (e.g. Opistotonus and convulsion) 60 neonate (35.5%) need exchange transfusion in addition to intensive phototherapy and the others need just intensive phototherapy. Cause of severe hyperbilirubinemia was ABO and Rh incompatibility (5.9%), G6PD deficiency (25.5%), sepsis (12%) other causes such as spherocytoses and immune hemolytic anemia (3.5%) and unknown (53.1%) (Figure 1). Risk factor of hyperbilirubinemia in our patients were 1) Male sex (P = 0.017), 2) previous siblings with severe hyperbilirubinemia (P = 0.006), 3) early discharge (P = 0.035), 4) NVD (P = 0.027) (it may be due to early discharging of neonates with NVD than cesarean section), 5) Brest feeding(P =0.038) and 6) concept of using herbal medicine instead of referring to Doctor when neonate had Icter (P = 0.024).

**Table 1.** Cause of Hyperbillirubinemia in Patients and Their Siblings

Cause of Hyperbillirubinemia	Patients, %	Siblings, %
**Sepsis**	12	11.4
**G6PD deficiency**	25.5	30.7
**ABO/RH**	5.9	5.8
**Others**	3.5	2.1
**Unknown**	53.1	50

## 5. Discussion

In our study, the prevalence of severe hyperbilirubinemia was 15 percent in all neonates with Icter. Also, it is more prevalent in males. FOK et al. reported that severe hyperbilirubinemia had 23.9% prevalence in China (16). Other studies showed prevalence of about 8-17 % (11-14). The difference may be due to definition of severe hyperbilirubinemia that FOK included all neonates with bilirubin of more than 12 mg/d/lit. Also Mirfazeli et al. reported that prevalence of hyperbilirubinemia was 12% in north of Iran (17) Gilbert syndrome (Gs) is a common entity in Iran probably due to the higher number of consanguineous marriages in Fars province, the method of screening (rifampin test), and the high genetic susceptibility in Iran. In our previous studies, we showed that GS alone cannot cause severe indirect hyperbilirubinemia, but it may have a summative effect to increase bilirubin when combined with other factors, for example, G6PD deficiency, and other unknown problems. Our results showed that in GS males are affected about twice as often as females; however, the cause of this preponderance is unknown and needs further investigation. Also, GS was found to be more prevalent in Iran (18, 19). It may be the cause of more prevalence severe hyperbilirubinemia in Fars province. In our study, the cause of severe hyperbilirubinemia, similar to other previous studies in the world, was ABO and Rh incompatibility (5.9%), G6PD deficiency (25.5%), sepsis (12%) other causes (3.5%) and unknown (53.1%).Causes of severe hyperbilirubinemia in our study were 53.1% unknown. In comp bell study in 70% of cases, the cause of hyperbilirubinemia was unknown. Other studies showed that 20-70% of cases are unknown. In Sgro study in Canada, the cause for severe hyperbilirubinemia was identified in 36.0% of cases. ABO blood group incompatibility was the most common cause, followed by G6PD deficiency .Of those with ABO incompatibility, 66.7% involved infants born to mothers with type O blood. In 64.0% no cause was identified for the hyperbilirubinemia (15). It means that causes of severe hyperbilirubinemia in the entire world are similar, but more investigations need to be done to find out the unknown causes. In our study, risk factors of severe hyperbilirubinemia was detected as 1) Male sex , 2) previous siblings with severe hyperbilirubinemia, 3) early discharge, 4) NVD (it may be due to early discharging of neonates with NVD than cesarean section), 5) Breast feeding and 6) concept of using herbal medicine instead of referring to Doctor when neonate had Icter. But the level of gradual education of mothers, maternal age, and cephal hematoma that was mentioned as risk factors of severe hyperbilirubinemia in previous studies (1, 3, 13, 15, 20-22) was not increased risk of severe hyperbilirubinemia in our study and it showed that ethnic and cultural background of the mothers was more important than school education in our country. In our study, in spite of careful screening 2 cases developed Kernicterus before referring to doctor, so it showed importance of global education via media. Our study showed that severe neonate indirect hyperbilirubinemia is still prevalent, although many programmed screening for hyperbilirubinemia also exists and cause of more than half of cases is still unknown. In our study, risk factors of severe neonatal hyperbilirubinemia was 1) male sex, 2) previous siblings with sever hyperbilirubinemia 3) early discharge 4) NVD 5) Breast feeding 6) cultural concept of using herbal medicine instead of referring to doctor when neonate had Icter. Our study showed in Fars province ethnic and cultural background of the mothers was more effective than school education in preventing hyperbilirubinemia complication.
